# HyperSight CBCT dose calculation accuracy for adaptive head and neck radiotherapy: Results from a prospective clinical trial

**DOI:** 10.1002/mp.70632

**Published:** 2026-08-06

**Authors:** Abby Yashayaeva, R Lee MacDonald, Kenny Zhan, Jennifer DeGiobbi, Natasha McMaster, Dave McAloney, Lucy Ward, Cheryl Anderson, Marc LeBlanc, Lara Best, Murali Rajaraman, Derek Wilke, Amanda Cherpak

**Affiliations:** ^1^ Department of Physics and Atmospheric Sciences Dalhousie University Halifax Nova Scotia Canada; ^2^ Department of Radiation Oncology Dalhousie University Halifax Nova Scotia Canada; ^3^ Nova Scotia Health Halifax Nova Scotia Canada

**Keywords:** adaptive radiotherapy, cone beam computed tomography, metal artifact reduction

## Abstract

**Background:**

Head and neck (H&N) patients frequently undergo anatomical changes such as weight loss and tumour shrinkage during treatment, requiring repeat imaging and replanning to maintain accurate dose delivery. On‐board cone‐beam CT (CBCT) systems could support these workflows, however they have historically been limited by reduced Hounsfield unit accuracy, inferior image quality, and susceptibility to metal artifacts from dental hardware, all of which are critical for effective radiotherapy treatment planning and dose delivery. HyperSight CBCT offers improved image quality comparable to FBCT along with metal artifact reduction techniques, but its dosimetric performance in H&N patients, particularly in the presence of dental metal implants, has not yet been evaluated.

**Purpose:**

This work evaluates the dosimetric accuracy of the HyperSight CBCT system (Varian Medical Systems), including the impact of metal artifact reduction, to determine its reliability for H&N treatment planning.

**Methods:**

Images of 25 H&N cancer patients were acquired on a conventional fan‐beam CT (FBCT. GE Healthcare) and on two CBCT systems: an Ethos radiotherapy system equipped with HyperSight CBCT, which provides advanced iterative reconstruction (HS‐Acuros) and metal artifact reduction reconstruction (HS‐MAR) options, and a TrueBeam linear accelerator system with conventional CBCT, which uses filtered back projection reconstruction (Varian Medical Systems). For each patient, the CBCTs were rigidly registered to the FBCT, and the clinically accepted reference plans were forward calculated on all images. Gamma evaluations (3%/3 mm, 2%/2 mm, 1%/1 mm) for all CBCT modes were performed relative to FBCT on slices both unaffected and affected by metal artifacts, with the latter also analyzed as a function of the metal artifact index (AI). DVH metrics were assessed for the planning target volume (PTV), mandible, oral cavity, and parotid glands.

**Results:**

For all gamma criteria, HS‐Acuros images demonstrated significantly higher pass rates than both HS‐MAR and TrueBeam CBCTs in the artifact‐free slices (*p* ≤ 0.033). In artifact‐affected slices, HS‐MAR reconstructions performed the best in slices affected by metal artifacts (*p* ≤ 0.001), exhibiting the least reduction in gamma pass rates with increasing artifact index (AI). Conventional TrueBeam CBCT consistently yielded the lowest gamma pass rates in artifact‐prone regions (*p* ≤ 0.005). Across all slices, the median 3D 3%/3 mm gamma passing rates were above 99.2%, 98.7% and 97.7% for HS‐Acuros, HS‐MAR and TrueBeam, respectively. Across all CBCT images, the median DVH‐metric deviations relative to CTsim FBCT were within 2% for all structures and metrics except for PTV Dmax.

**Conclusions:**

HyperSight provides imaging suitable for direct dose calculation in H&N adaptive radiotherapy with iCBCT MAR improving accuracy in the presence of dental metal artifacts; however, iCBCT Acuros may be preferred when artifact reduction is not critical. This work highlights a significant advancement towards CBCT‐based direct‐dose calculation for adaptive radiotherapy.

## 1 INTRODUCTION

The dynamic approach of adaptive radiotherapy enables adjustments to the patient's plan during treatment to account for changes in anatomy.^1^, ^2^ Such adaptation is particularly relevant for head and neck (H&N) cancer patients, who often experience significant anatomic changes, including weight loss and tumor shrinkage during the course of radiation therapy.^1^, ^3^, ^4^ If substantial volume changes occur after treatment begins, patients may require a second fan‐beam CT (FBCT) scan and the creation of a new treatment plan^4^, ^5^, ^6^ to maintain accurate dose delivery. This offline re‐planning process can take several days to weeks during which patients may either continue receiving treatment based on the original plan, or the treatment may be paused until a new plan is created.^7^ There is an increasing demand for on‐board cone‐beam CT (CBCT) scanners to provide image quality comparable to that of a FBCT.^8^ This can enable the clinical implementation of online adaptive radiotherapy with precise treatment planning and dose calculations directly at the treatment unit, as well as support offline adaptive workflows by reducing the need for repeat FBCT imaging.

Historically, CBCT images have had inferior image quality and Hounsfield unit (HU) accuracy compared to FBCT,^9^ limiting their suitability for tumour and organ delineation and dose calculations necessary for treatment plan adaptation.^10^, ^11^, ^12^, ^13^ To overcome this, a synthetic CT (sCT) is commonly generated by deforming the HU values from the FBCT onto the anatomy captured in the CBCT.^14^, ^15^ However, uncertainties in deformable image registration along with differences in tissue densities captured between the FBCT and CBCT can lead to inaccurate sCTs, which in turn introduce uncertainty into calculated dose distributions.^16^, ^17^

An additional challenge in H&N patients arises from the presence of dental implants or fillings, which can produce severe artifacts in CT imaging, leading to distorted HU values and obscured surrounding anatomy.^18^, ^19^ These artifacts can compromise target delineation and propagate into dose calculation errors,^20^, ^21^ increasing the risk of tumor underdosing or unnecessary irradiation of nearby normal tissues. To address this issue, metal artifact reduction (MAR) algorithms have been developed to improve CBCT image quality in the presence of high‐density materials.^22^

The HyperSight imaging solution is available as an option on the various Varian linacs, including the TrueBeam, Halcyon and Ethos radiotherapy systems.^23^ In this study, HyperSight CBCT images were acquired on an Ethos adaptive radiotherapy system, while conventional CBCT images were acquired on a standard TrueBeam linear accelerator system. For Halcyon and Ethos, HyperSight features a large‐area detector (86 cm × 43 cm) with a cesium iodide (CsI) scintillator, scatter‐reducing grid, low‐noise electronics, and rapid image acquisition (up to 70 frames per second). HyperSight provides multiple image reconstruction methods, including iCBCT Acuros, which incorporates scatter correction and iterative reconstruction techniques, and is recommended for achieving the highest accuracy of Hounsfield Unit (HU) values,^24^, ^25^, ^26^ and iCBCT MAR, an iterative reconstruction approach for reducing metal artifact. The technical advances of HyperSight have been shown to produce CBCT images with quality superior to previous CBCT platforms,^24^ and HU accuracy comparable to standard FBCT images,^27^, ^28^ even in the presence of high‐density materials when using MAR reconstruction.^29^, ^30^, ^31^ Building on these findings, the next critical question is whether the improved image quality of HyperSight CBCT translates into accurate dose calculations. Inaccurate HU values, image non‐uniformities, or artifacts could lead to systematic dose errors, affecting both target coverage and organ‐at‐risk (OAR) sparing. A robust dosimetric validation is therefore essential to validate the implementation of HyperSight in adaptive radiotherapy workflows.

Recent studies have investigated the use of HyperSight CBCT for direct dose calculation across multiple anatomical sites including liver, lung, breast, abdomen, chest wall, pelvis, prostate, and pancreatic regions, showing accuracy comparable to that of both sCTs and planning CTs.^32^, ^33^, ^34^ However, these findings have not yet been validated in a large H&N patient cohort. Additionally, the impact of clinically relevant dental metal artifacts, commonly encountered in H&N patients, on HyperSight CBCT‐based dose calculation has not been systematically investigated. The present study addresses these gaps by evaluating the dosimetric performance of HyperSight CBCT through a comparative analysis of treatment plans calculated on HyperSight iCBCT Acuros, HyperSight iCBCT MAR, conventional TrueBeam CBCT, and CTsim FBCT images in H&N radiotherapy patients. In particular, it provides the first clinical evaluation of Hypersight iCBCT MAR reconstruction for dose calculation in the presence of dental metal artifacts. To our knowledge, it also represents the first clinical assessment of the feasibility of HyperSight for CBCT‐based dose calculation in adaptive radiotherapy conducted on a large‐population H&N patient dataset. This work builds upon our previously published image quality analysis of the same patient cohort.^35^

## 2 METHODS

### 2.1 Ethical approval and participants

This prospective study was registered on ClinicalTrials.gov (Identifier: NCT05666193) on July/07/2023 and received ethics approval from the Research Ethics Board (REB File#: 1028935) on February/06/2023. Eligible participants were adults (≥19 years) undergoing curative‐intent radiation therapy for head and neck cancer on a TrueBeam system, receiving at least 21 treatment fractions. Recruitment and data collection took place between August 2023 and October 2024, and 25 patients were included in the analysis, prescribed 70 Gy in 35 daily fractions. Within this cohort, 17 had oropharyngeal disease, 2 had tonsillar tumors, 1 had hypopharyngeal involvement, 1 had nasopharyngeal disease, and 4 had laryngeal tumors. Written informed consent was obtained from all participants prior to enrollment, and each patient was assigned a study‐specific identifier to ensure the anonymization of all collected data. Further details can be found in previously published work from this clinical study.^35^, ^36^

### 2.2 Image acquisition

Patients were positioned on the treatment couch using a thermoplastic immobilization mask. Planning images were acquired on a GE Optima 580 RTCT CT simulator (CTsim FBCT, GE Healthcare)^35^ with Feldkamp‐Davis‐Kress (FDK) reconstruction, which did not have MAR capabilities, and on a TrueBeam system^23^ (version 2.7 MR4) with conventional CBCT using FDK reconstruction, as part of standard clinical workflow for treatment planning and patient positioning, respectively. HyperSight CBCT acquisition was performed only as part of the clinical trial for image analysis and is not part of our standard clinical workflow. HyperSight CBCT images were acquired on the Ethos platform reconstructed with iCBCT Acuros on the same day as the CTsim FBCT, using the longitudinal extended field of view acquisition mode, spanning 38 cm from the top of the head to the lungs. The TrueBeam CBCT scans were acquired on the first day of treatment, with the interval between simulation and treatment ranging from 12 to 20 days. Additional HyperSight and TrueBeam CBCTs were collected at fraction 21 as part of a clinical trial evaluating adaptive workflows on Ethos. Since these later scans were acquired several weeks after the initial FBCT images, fraction 21 images were excluded from the current analysis, and evaluation of anatomical changes over time and the implications for plan adaptation will be reported in future work. The acquisition protocols are outlined in Table 1, as previously reported in an image quality analysis performed on the same patient cohort.^36^

**TABLE 1:** Imaging protocol parameters used for image acquisition in the study.

	CTsim FBCT	TrueBeam CBCT	HyperSight CBCT
Exposure (mAs)	32 – 69	150 – 151	174 – 350
x‐ray tube voltage (kVp)	120	100	125
Slice thickness (mm)	2.5	2	2
Pixel size (mm)	1.27	0.51–0.91	1.37
CTDIvol (mGy)	22 – 26	3.17–3.20	3.94–3.95
Axial FOV (mm)	67	26–46	31 –72
Longitudinal FOV (mm)	45 – 52	19–34	38

### 2.3 Preprocessing

The raw projection data from the Ethos system were extracted and externally processed to generate an additional HyperSight image dataset using the iCBCT MAR reconstruction mode. All image sets were imported into the Eclipse treatment planning system (version 18.0, Varian Medical Systems) and aligned to the CTsim FBCT through rigid image registration. The treatment planning target volume (PTV) and organ at risk (OAR) structures defined according to institutional standards on the FBCT were propagated onto the other image sets and subsequently verified and adjusted by dosimetrists to account for minor discrepancies in patient setup. Areas affected by streak artifacts from high‐Z dental materials were contoured and manually overridden to HU = 0 to approximate soft‐tissue density on the FBCT images only, following standard artifact‐mitigation practices and to support dose comparisons relative to the FBCT baseline. No additional post‐processing or artificial modifications were performed on the CBCT images to allow a comparison between a MAR‐enabled workflow (HyperSight iCBCT MAR) and non‐MAR workflows (HyperSight iCBCT Acuros and conventional TrueBeam CBCT).

### 2.4 Hounsfield unit to mass density verification

The Ethos Treatment Management system allows implementation of a custom mass density (MD) calibration curve. At our center, a calibration curve measured using an Advanced Electron Density (AED) phantom^36^ is clinically implemented for both offline and online dose calculations. To verify the relationship between MD and HU, we acquired scans of the AED phantom containing tissue‐equivalent cylindrical inserts with mass densities ranging from lung (0.29 g/cm^3^) to cortical bone (1.93 g/cm^3^) on the CTsim FBCT, TrueBeam, and HyperSight reconstructed with iCBCT Acuros and iCBCT MAR. The same phantom configuration, setup was used for all image acquisitions, and the same imaging protocols as those used for patient imaging were applied. For each insert, HU values were averaged within cylindrical volumes of interest (2 cm in diameter, spanning three slices) centered on the insert. The relationship between MD and HU was plotted for each imaging modality and compared to CTsim FBCT using mean absolute deviation (MAD) and maximum deviation metrics. These results were further validated against previously published CBCT mass density calibration data,^33^, ^34^ supporting the suitability of using a common calibration curve.

### 2.5 Dose calculation

The VMAT reference plans were copied and forward calculated on all images using the Acuros XB 15.6 dose calculation model,^23^ with a calculation grid size of 0.2 × 0.2 cm^2^ and dose reported to medium. The same HU to MD calibration curve, clinically implemented for the CTsim FBCT at our center, was applied to all image types. High‐Z materials exceeding the range of the clinical calibration curve were segmented and assigned overrides of HU = 3083 and mass density = 3 g/cm^3^ prior to dose calculation.

### 2.6 Dose calculation analysis

#### 2.6.1 Gamma evaluation

**FIGURE 1:**
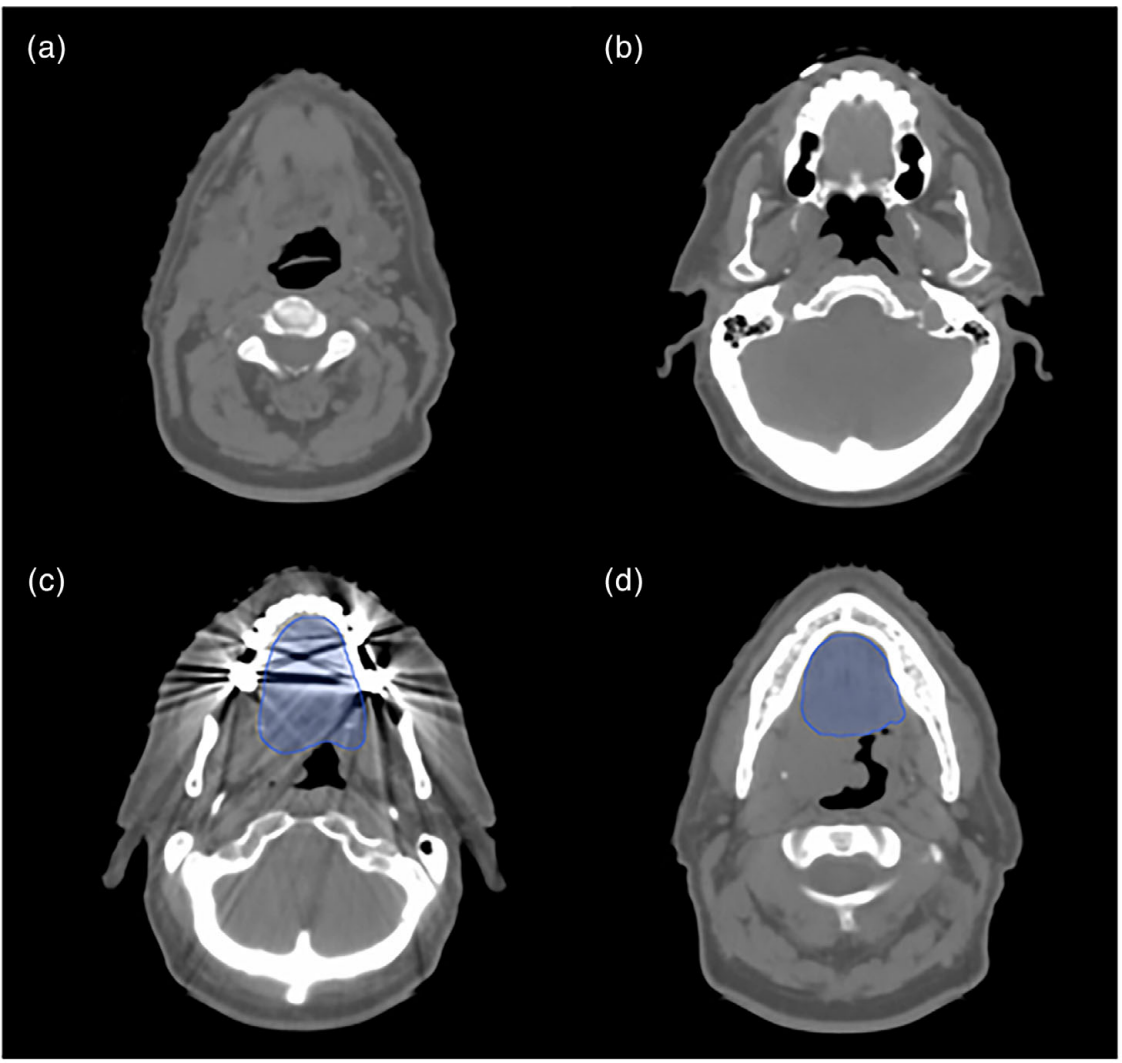
Axial slices from a CTsim FBCT scan of a single patient used for the gamma evaluations: (a) below the mandible, (b) above the oral cavity, (c) within the oral cavity at the location of maximum metal artifact, and (d) within the oral cavity at a location with minimal artifact. The artifact and background ROIs, outlined in blue in (c) and (d), were used to calculate the artifact index (AI).

Gamma evaluations between the three CBCT images and the FBCT, which served as the reference, were performed for each patient on multiple slices to evaluate the forward‐calculated dose distributions in different anatomical regions. Three representative axial slices were selected consistently across all patients for this analysis: (a) the slice directly below the mandible containing predominantly soft tissue, (b) the slice directly above the oral cavity containing high‐density bone and low‐density air gaps, and (c) the slice most affected by metal artifacts in the oral cavity, or if no significant artifacts were present, the central slice of the oral cavity. Figure 1a–c illustrate the corresponding slices (a), (b), and (c) from the CTsim FBCT image for one representative patient. Both 2D and 3D gamma index calculations use slice‐by‐slice comparisons between two dose distributions. In contrast to 2D analysis, the 3D gamma evaluation incorporates dose information from neighboring slices within the volumetric dataset. For slices (a) and (b), a 3D gamma evaluation was performed to determine the dose accuracy among normal tissues situated away from metal artifacts. This provided a baseline assessment of unaffected regions. For slice (c), a 2D evaluation was used to more accurately capture localized metal artifacts, which were typically confined to a few slices with limited longitudinal extent. Passing criteria of 3%/3 mm, 2%/2 mm, and 1%/1 mm (dose difference/distance‐to‐agreement) were applied with a 10% dose threshold relative to the global maximum dose, such that voxels receiving less than 10% of the maximum dose were excluded from the gamma analysis. The Wilcoxon signed‐rank test *p*‐values were used to compare paired results between CBCT image sets. Corresponding *p*‐values were used to assess whether differences between modalities were statistically significant, with *p* < 0.05 considered statistically significant.

#### 2.6.2 Artifact index

Metal artifacts were observed in 22 of 25 patients, primarily associated with dental implants and fillings. These appeared as bright and dark streaks that overlapped critical anatomical structures, including the PTV and oral cavity. To evaluate the impact of metal implants on dose accuracy, the artifact index (AI), defined in Equation 1, was measured on the CTsim FBCT images, which did not include MAR capabilities.

(1) $$AI=\sqrt{SD{\left(ROI_{artifact}\right)}^{2}-SD{\left(ROI_{background}\right)}^{2}}\;$$

where $ROI_{artifact}$ and $ROI_{background}$ are the oral cavity contours in the slices exhibiting the highest and lowest pixel standard deviations (SD), outlined in blue in Figure 1c and d, respectively. Notably, $ROI_{artifact}$​ is the oral cavity structure located on the same slice as slice (c). The TrueBeam CBCT, HyperSight iCBCT Acuros and HyperSight iCBCT MAR gamma passing rates for slice (c) were analyzed as a function of the AI calculated for the corresponding CTsim FBCT image.

#### 2.6.3 Dose‐volume histogram metrics

Dose‐volume histogram (DVH) metrics were evaluated for the PTV (D2%, D95%, D99%, Dmean, Dmax, V95%, V99%, and V100%), the mandible (D2%, Dmean, Dmax, V50% V60% and V70%), the oral cavity (D2%, Dmean, Dmax, V60%, V80%, V90%, and V95%) and the left and right parotid glands (D2%, Dmean, Dmax, V30%, V50% and V80%). The selected volume‐based metrics were chosen near the steepest regions of each DVH where small dose variations have the greatest clinical impact. Due to the limited axial field of view of the TrueBeam CBCT system, portions of the surrounding thoracic anatomy were truncated in some patient images. In 11 cases, the missing anatomy near the inferior edge of the PTV led to inaccuracies in dose calculation, resulting in apparent PTV hotspots in the TrueBeam images. The OARs were located more superiorly within the imaging field and were therefore not affected by the truncation. Consequently, TrueBeam images for 14 patients were included in the PTV DVH analysis, while all 25 TrueBeam Images were included in the OAR analyses. Differences in DVH metrics relative to CTsim FBCT, defined as $\Delta \mathrm{DV}H_{M}$ in Equation 2, were used to assess dosimetric accuracy. In this equation, $\mathrm{DV}H_{M,i}$ represents the metric from the image under evaluation, e.g. $\mathrm{DV}H_{M,\mathrm{iCBCT}\;\mathrm{Acuros}}$, and $\mathrm{DV}H_{M,\mathrm{FBCT}}$ represents the corresponding metric from the CTsim FBCT image.

(2) $$\Delta \mathrm{DV}H_{M}=\mathrm{DV}H_{M,i}\;\;-\;\mathrm{DV}H_{M,\mathrm{FBCT}}$$

## 3 RESULTS

**FIGURE 2:**
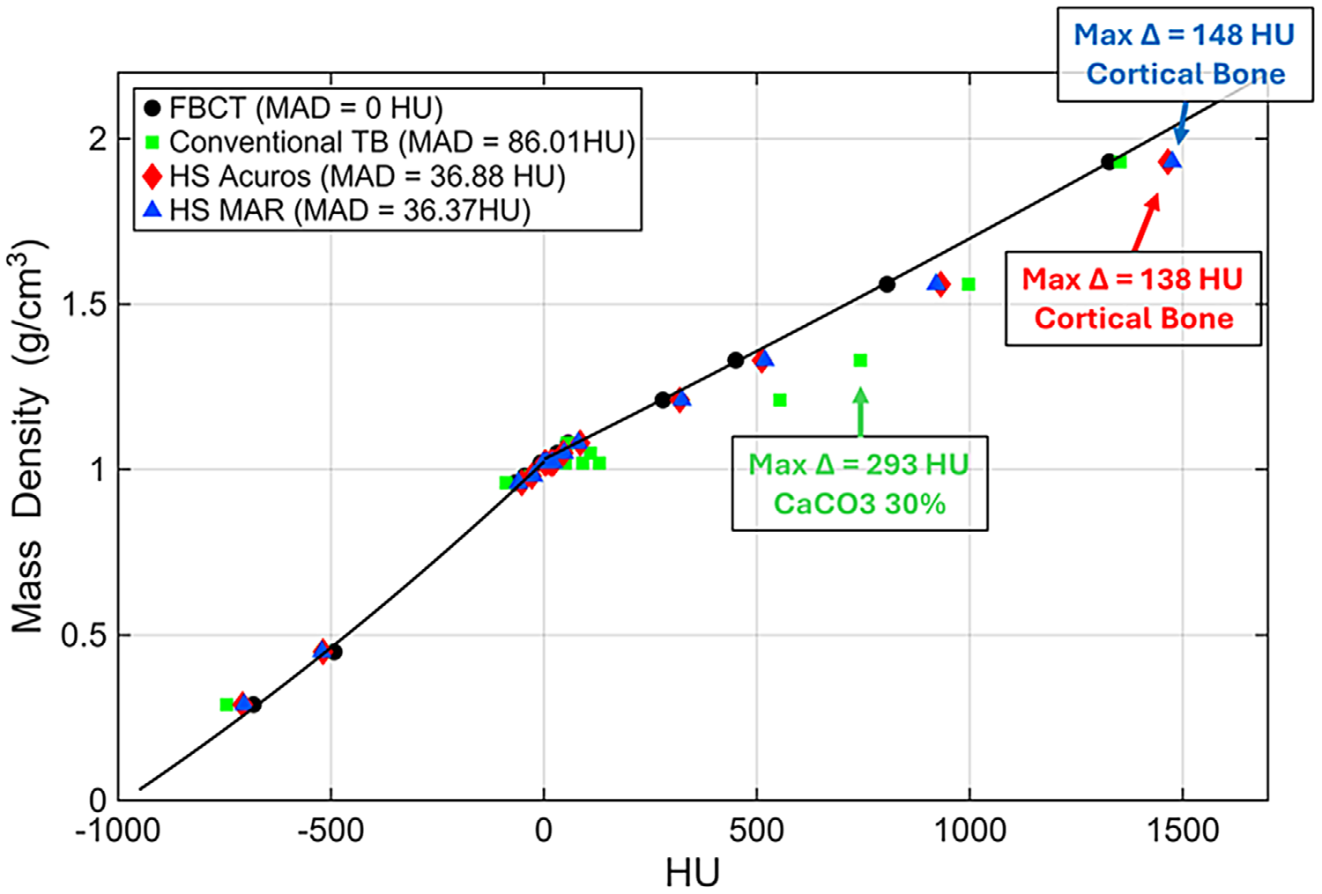
HU to mass density measurements from an AED phantom acquired on a CTsim FBCT, conventional TrueBeam CBCT, HyperSight iCBCT Acuros and HyperSight iCBCT MAR. For each imaging modality the mean absolute deviation (MAD) values, calculated as the average absolute difference relative to the CTsim FBCT across all tissue‐equivalent inserts, are presented in the legend. The inserts with maximum deviations from CTsim FBCT are indicated with arrows.

Before analyzing patient dose distributions, the relationship between MD and HU was examined for each CBCT modality to validate the interpretation of subsequent patient‐specific dose comparisons. In Figure 2, the MD values are plotted against the HU values measured for each tissue‐equivalent insert in the AED phantom. Across all density ranges, the mean absolute deviation values relative to CTsim FBCT were 86.0 HU for conventional TrueBeam, 36.9 HU for HyperSight iCBCT Acuros, and 36.4 HU for HyperSight iCBCT MAR. The maximum deviations were within 293 HU for TrueBeam, 138 HU for HyperSight Acuros, and 148 HU for HyperSight MAR. Six of the rods in the phantom were water‐equivalent inserts. For these inserts, the mean absolute HU deviation relative to CTsim FBCT was 64.7 HU for conventional TrueBeam CBCT, 16.7 HU for HyperSight iCBCT Acuros, and 14.7 HU for HyperSight iCBCT MAR.

**FIGURE 3:**
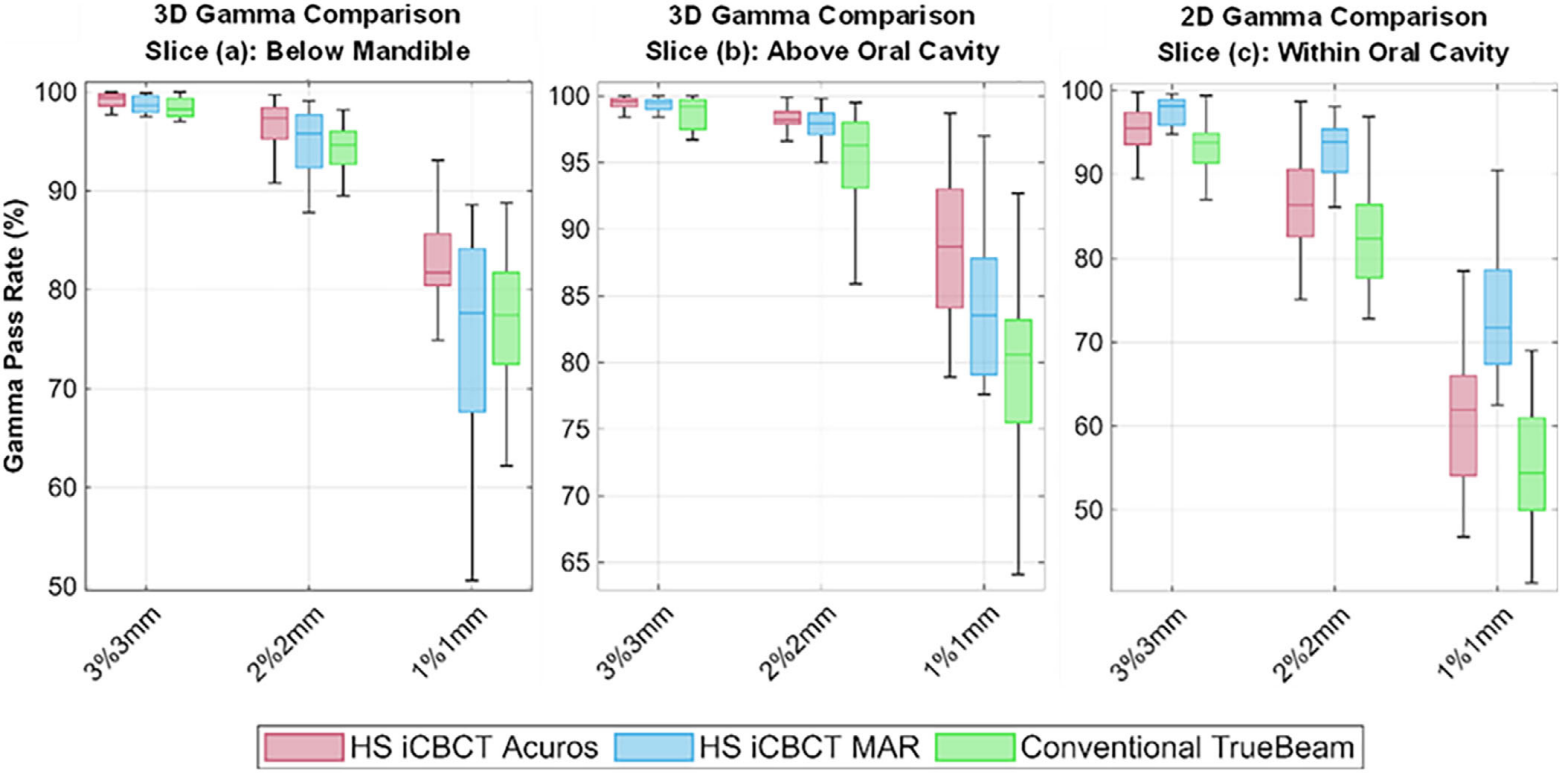
Gamma pass rate values comparing dose distributions of VMAT H&N treatment plans calculated on HyperSight iCBCT Acuros, HyperSight iCBCT MAR, and TrueBeam CBCT, to the reference CTsim FBCT image, for 25 patients. The median, 25th and 75th percentiles, and the minimum and maximum values are shown for (a) a slice below the mandible containing mostly soft tissue, (b) a slice above the oral cavity with regions of high‐density (bone) and low‐density (air gaps), and (c) the slice most affected by metal artifacts within the oral cavity, or, if no significant artifacts were present, the central slice of the oral cavity.

For the H&N patient cohort, the forward‐calculated CBCT dose distribution analysis highlights the impact of imaging modality and artifact severity on dose accuracy. The 3%/3 mm, 2%/2 mm, and 1%/1 mm gamma pass rates for the three evaluated slice locations are shown in Figure 3. For slice a), a region below the mandible containing predominantly soft tissue, the 3D gamma pass rates of the HyperSight iCBCT Acuros images were significantly higher than those of HyperSight iCBCT MAR and TrueBeam CBCT images across all three passing criteria (*p* ≤ 0.033). For the clinically common 3%/3 mm criterion, the median 3D gamma pass rates (± interquartile range) were 99.4% ± 1.0% for HyperSight iCBCT Acuros, 98.7% ± 1.6% for HyperSight iCBCT MAR, and 98.3% ± 1.8% for TrueBeam CBCT. For slice b), a region above the oral cavity containing high‐density bone and low‐density air gaps, the 3D gamma pass rates of the HyperSight iCBCT Acuros images remained significantly higher across all passing criteria (*p* ≤ 0.004). For the 3%/3 mm criterion, the median 3D gamma pass rates were 99.6% ± 0.6%, 99.5 ± 0.7% and 99.2% ± 2.1% for HyperSight iCBCT Acuros, HyperSight iCBCT MAR, and TrueBeam CBCT, respectively. For slice c), the slice most affected by metal artifacts in the oral cavity (or the central oral cavity slice if no significant artifacts were present), the 2D gamma pass rates of the HyperSight iCBCT MAR images were significantly higher than both other image types across all passing criteria (*p* ≤ 0.001), while TrueBeam CBCT exhibited the lowest pass rates (*p* ≤ 0.005). For the 3%/3 mm criterion, the median 2D gamma pass rates were 95.5% ± 3.7%, 98.2 ± 3.0%, and 93.8% ± 3.4% and the 3D gamma pass rates were 99.2% ± 1.5%, 99.2% ± 1.2% and 97.7% ± 2.9 % for HyperSight iCBCT Acuros, HyperSight iCBCT MAR, and conventional TrueBeam CBCT, respectively. The 2%/2 mm and 1%/1 mm criteria demonstrated the same overall trends and relative behavior between CBCT modalities.

**FIGURE 4:**
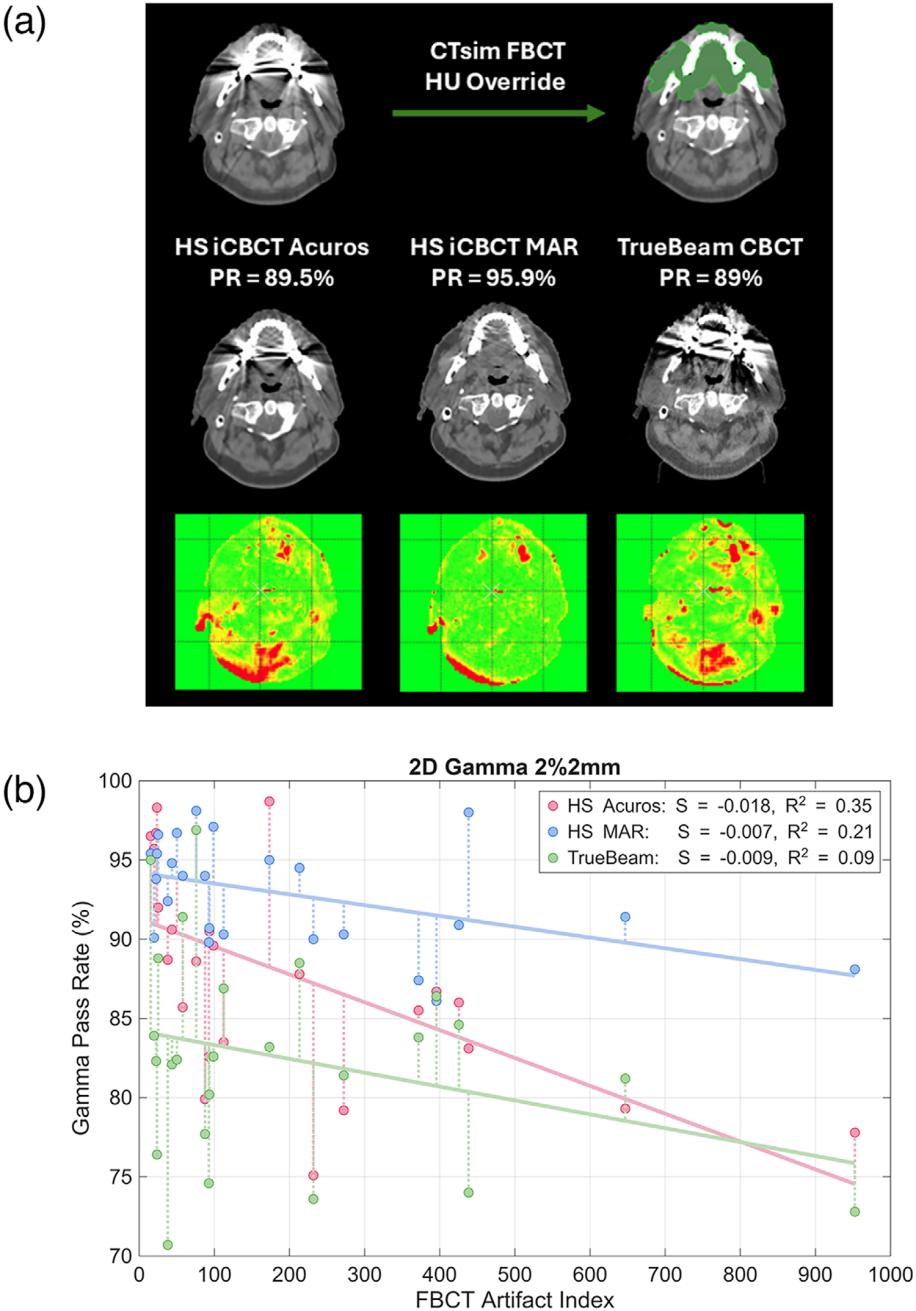
(A) Axial slices at location (c)—the slice in the oral cavity most affected by metal artifacts, for one representative patient (artifact index on FBCT = 231.77), shown using the same window width and level settings. The artifact contour used for the HU override is outlined in green on the CTsim FBCT image. The 2%/2 mm 2D gamma distributions, calculated using the HU‐overridden CTsim FBCT as the reference, are shown below each image and quantified using the gamma pass rates (PR). (b) The 2%/2 mm 2D gamma pass rates plotted as a function of the artifact index computed from the original CTsim FBCT images for all patients, with the slope (S) and coefficient of determination (*R* ^2^) of the linear fits indicated in the legend.

Figure 4a shows the 2%/2 mm 2D gamma distributions for HyperSight and conventional TrueBeam CBCT images for a representative patient on slice (c). The deviations in the right‐posterior neck correspond to low‐dose regions that can be sensitive to minor discrepancies, likely due to small positioning differences, voxel averaging, or other low‐dose effects. In the oral cavity, deviations in the HyperSight iCBCT Acuros and TrueBeam CBCT images can be explained by severe metal artifacts, which were substantially reduced in HyperSight iCBCT MAR images. In Figure 4b, the gamma pass rates are plotted against the artifact index computed from the original CTsim FBCT image, as the 2%/2 mm criterion is more sensitive to dose discrepancies and better highlights the effect of metal artifacts on dose accuracy. The slope (S) and coefficient of determination (*R* ^2^) of the linear fits are shown in the legend.

Despite the relatively low coefficient of determination (*R* ^2^ = 0.35), the HyperSight iCBCT Acuros images were most sensitive to artifact severity, showing the steepest decline in gamma pass rates with increasing artifact index and a higher *R* ^2^ value than for the other CBCTs. The HyperSight iCBCT MAR images yielded the highest gamma pass rates and the least negative slope with increasing artifact index, although several low‐artifact cases showed higher gamma pass rates with HyperSight iCBCT Acuros reconstruction. The TrueBeam CBCT images generally presented the lowest gamma pass rates across all artifact levels. Similar results were observed for the 3%/3 mm and 1%/1 mm gamma passing criteria.

**FIGURE 5:**
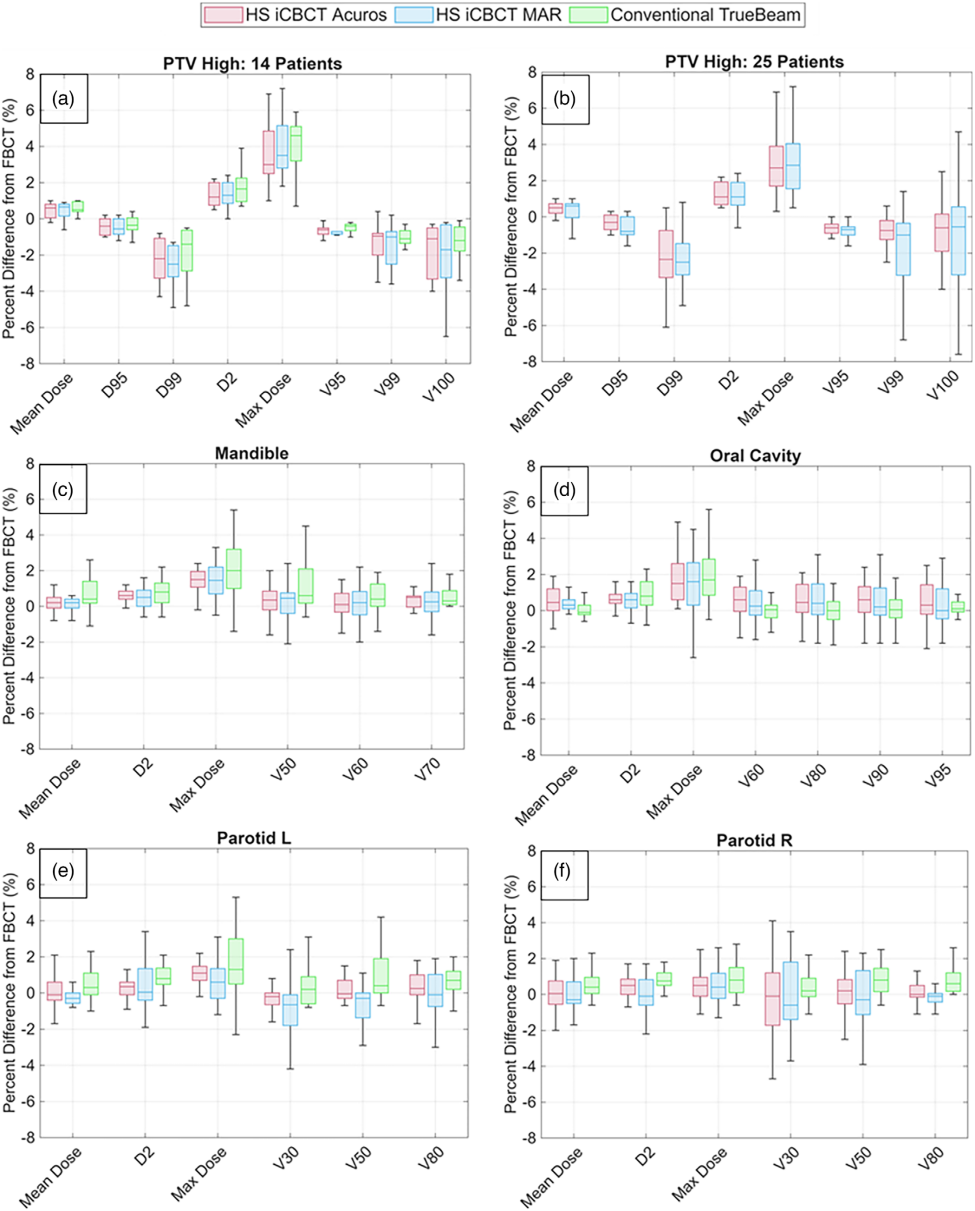
Deviations in DVH metrics relative to the CTsim FBCT for the PTV and OARs, showing the median, 25th and 75th percentiles, and the minimum and maximum values across (a) 14 patients with full PTV inclusion in the TrueBeam imaging FOV and (b–f) all 25 patients.

Figure 5 shows the differences in dosimetric parameters relative to CTsim FBCT for the PTV and various OAR structures. Figure 5a presents the PTV DVH metric comparison for all three CBCT datasets with 11 patients excluded due to truncation of the TrueBeam image at the inferior edge of the PTV. Figure 5b includes all 25 patients but compares only the HyperSight iCBCT Acuros and iCBCT MAR reconstructions. The largest median deviations (± interquartile range) were observed for the PTV Dmax metric in Figure 5a, reaching up to 3.0% ± 2.4% for HyperSight iCBCT Acuros, 3.5% ± 2.4% for HyperSight iCBCT MAR, and 4.6% ± 1.9% for TrueBeam CBCT. Median deviations for the PTV D2% metric were 1.2% ± 1.3%, 1.3% ± 1.2%, and 1.7% ± 1.3%, respectively. The OARs showed comparable deviations across images, with median DVH‐metric differences remaining within 2.0% and no CBCT modality consistently performing better or worse.

## 4 DISCUSSION

HyperSight CBCT images have the potential to support dose calculation and adaptive re‐planning for head‐and‐neck radiotherapy, which would reduce the need for additional CTsim scans and improve the efficiency and accuracy of treatment. Adaptive re‐planning is particularly important for H&N patients, who commonly experience anatomical changes due to tumour response and/or weight loss, and may need plan adjustments. Additionally, dental implants and other high‐density materials in the oral cavity can produce metal artifacts that obscure target volumes and OARs, degrade image quality, and compromise the accuracy of dose calculations. This study presents a comparative evaluation of the dosimetric accuracy in VMAT H&N treatment plans calculated on HyperSight images acquired on an Ethos machine using iCBCT Acuros and iCBCT MAR reconstructions, and conventional TrueBeam CBCT used for image‐guidance, relative to CTsim FBCT—the standard for treatment planning.

The HU deviations from CTsim FBCT observed in the MD‐HU values were consistent with those reported by Duan et al., who observed maximum deviations of approximately 200 HU for HyperSight images acquired at 125 kV.^34^ Although Duan et al. applied separate calibration curves for dose calculation, they implied that a calibration curve derived from planning CT scans could be used for HyperSight dose calculation if desired. Similarly, Sijtsema et al. concluded that the implementation of a dedicated calibration curve is not required to achieve accurate dose calculation.^33^ The MD‐HU analysis in our study revealed that for the HyperSight images, the larger deviations (approximately 140 HU) were observed primarily for the high‐density inserts (∼1000 HU or greater) which are typically present in small volumes. The TrueBeam images exhibited maximum deviations across a broader range of inserts, closer to common HUs found in patient scans. In our study, a single HU‐to‐mass density calibration curve derived from the CTsim FBCT was applied across all image types. This approach was chosen because implementing a separate calibration curve for the TrueBeam CBCT was not feasible due to the high level of artifacts and HU variability present in the phantom, which would compromise the reliability of the curve. HyperSight CBCT demonstrated a more consistent and reliable mapping of HU to mass density, which is critical for accurate dose calculation. While a shared calibration curve may be beneficial in cases where it is not possible to accurately generate a separate one (e.g., due to poor image quality in TrueBeam scans), the impact of modality‐specific calibration under different clinical conditions, such as various beam qualities, scatter conditions, and patient anatomy, remain an important area for further investigation.

Gamma analyses in regions unaffected by metal artifacts (slices a and b) demonstrated that dose calculations on HyperSight iCBCT Acuros images consistently achieved higher pass rates than both HyperSight MAR and conventional TrueBeam CBCT. This suggests that iCBCT Acuros reconstruction provides more accurate representation of soft‐tissue and bone HUs in artifact‐free regions. However, in slices containing metal artifacts (slice c), the iCBCT MAR reconstruction outperformed both iCBCT Acuros and TrueBeam CBCT, demonstrating how MAR effectively mitigates localized HU distortions caused by dental fillings. These results suggest that while MAR improves the dosimetric accuracy in the presence of artifact‐producing metal implants, it may not always be the optimal default imaging mode; iCBCT Acuros may be preferred when artifact reduction is not a concern. TrueBeam CBCT demonstrated statistically significantly lower gamma pass rates than both of the HyperSight reconstructions in artifact‐affected regions, confirming its sensitivity to metal‐induced HU errors.

The analysis of gamma pass rates as a function of the artifact index further emphasizes the role of MAR in stabilizing dose accuracy for different levels of artifact severity. For HyperSight iCBCT MAR images, the higher gamma pass rates and the relatively flat slope of gamma pass rate versus artifact index in Figure 4b demonstrate greater robustness in dose calculations with increasing artifact severity. Conversely, HyperSight iCBCT Acuros was most sensitive to strong artifacts, with a clear trend of decreasing pass rates and highest *R* ^2^ value, and TrueBeam CBCT displayed the lowest baseline accuracy across all artifact levels. These results demonstrate that the choice of CBCT modality and reconstruction algorithm can meaningfully affect dose calculations, particularly in regions affected by metal artifacts.

Conventional TrueBeam CBCT reconstruction relies on FDK filtered back projection reconstruction, which assumes ideal projection data and does not explicitly model the complex scatter interactions occurring during CBCT acquisition. Consequently, scattered photons and beam hardening effects propagate through the reconstruction process, contributing to image artifacts, reduced HU accuracy and increased image nonuniformity. HyperSight iCBCT Acuros uses a deterministic solution of the linear Boltzmann transport equation to model photon transport and estimate scatter distributions prior to reconstruction,^25^, ^26^ combined with iterative reconstruction techniques. HyperSight iCBCT MAR incorporates an iterative algorithm in which projection data corrupted by high‐density materials are identified, categorized, and corrected to reduce streaking artifacts and HU distortions surrounding dental fillings. The advanced reconstruction algorithms reflect a system‐level improvement in HyperSight, where scatter modelling, iterative reconstruction, and optional MAR collectively contribute to improved image quality and dosimetric performance compared with conventional TrueBeam CBCT. The superior gamma performance of HyperSight iCBCT Acuros in artifact‐free regions, and HyperSight iCBCT MAR in regions containing severe metal artifacts can likely be explained by the interpolation and smoothing associated with MAR reconstruction, which may reduce anatomical detail in regions without metal artifacts. This likely explains the higher gamma pass rates observed for HyperSight iCBCT Acuros in artifact‐free regions, and HyperSight iCBCT MAR in regions containing severe metal artifacts. This effect was not observed in the AED phantom where the geometry and composition are comparatively uniform and lack the complex anatomical heterogeneity present in patient images. These findings are consistent with our previous image quality evaluation,^35^ in which iCBCT Acuros achieved image quality most comparable with FBCT in artifact‐free regions, while iCBCT MAR performed best in the presence of metal artifacts.

The PTV and OAR deviations in DVH metrics relative to CTsim FBCT were generally within clinically acceptable ranges across all CBCT images. For all structures, TrueBeam CBCT exhibited the largest median deviations in the D2% and Dmax metrics, indicating an overestimation of hot spots. The other DVH‐metrics were comparable across all images, with deviations typically within 2.5%. No CBCT modality consistently outperformed the others, suggesting that all evaluated CBCT systems can provide reasonably accurate dose estimates, as long as artifact mitigation is considered.

Previous studies have demonstrated that substantial HU uncertainties generally have a low impact on dose calculations.^37^, ^38^, ^39^ However, dose calculation accuracy alone does not fully determine the clinical adequacy of an image for treatment planning. Clear visualization of the target and surrounding organs is essential, as treatment plans are guided by their contours. TrueBeam CBCT, while sufficient for certain applications, presents several drawbacks. Its limited axial field of view excluded portions of the thorax in several patients, resulting in dose hotspots near the inferior edge of the PTV in 11 cases. Pronounced image artifacts in TrueBeam CBCT reduce image clarity and interfere with accurate contouring of targets and OARs. Without the ability to define boundaries between regions intended to receive high dose and those that should be spared, it is difficult to use these images to drive any re‐optimization of the treatment plan. In contrast, HyperSight CBCT has demonstrated improved HU accuracy^24^, ^27^, ^28^ and reduced sensitivity to metal artifacts,^29^, ^30^, ^31^ enabling clearer visualization of anatomy for accurate contouring. As reflected in the relatively small DVH differences observed between modalities, TrueBeam CBCT may provide acceptable dose estimates under favorable imaging conditions. However, this should be interpreted in the context of its imaging limitations, since conventional CBCT is susceptible to significant metal‐induced streaking artifacts and field of view truncation effects, which can compromise anatomical interpretation and reduce robustness for adaptive planning across a broader patient population. The central clinical takeaway of this work is that HyperSight addresses these practical limitations of TrueBeam CBCT, thereby supporting the clinical feasibility of CBCT‐based adaptive radiotherapy. The development and implementation of MAR techniques for TrueBeam CBCT may help mitigate some of these limitations, however in practice, edge cases would remain a clinical barrier. Future work investigating dose calculation accuracy on TrueBeam CBCT with MAR reconstruction would therefore be valuable to provide further understanding of its potential role in adaptive radiotherapy workflows.

Previous patient studies have examined dose distributions calculated on HyperSight images for other anatomical sites,^32^, ^33^, ^34^ however evaluations in the H&N region, particularly regarding dental related metal artifacts and MAR reconstruction, have been limited. A breath‐hold study by Martin et al.^32^ assessed the dosimetric accuracy of treatment plans calculated on HyperSight CBCT of patients with thoracic or upper abdominal cancer. The gamma scores of HyperSight iCBCT Acuros were statistically significantly higher than TrueBeam CBCT for all gamma criteria, and median DVH metric differences from corresponding CTsim values were within ± 2.3 % for HyperSight iCBCT Acuros and ± 2.7 % for conventional TrueBeam CBCT, respectively. Similarly, Duan et al.^34^ compared dose calculations on HyperSight iCBCT Acuros images with those on sCT for pelvic and breast patients and reported mean 3%/2 mm gamma pass rates of 99.57% for the sCT and 96.45% for HyperSight, using a 10% dose threshold. These studies did not include evaluation of the H&N region or dental related artifacts and therefore did not assess the iCBCT MAR reconstruction mode.

Sijtsema et al. evaluated dose calculation accuracy on HyperSight CBCT for prostate and lung cancer patients, reporting DVH metric differences within 2.1% for prostate and up to 20.3% for lung cases, the latter due to motion artifacts. The average 2%/2 mm gamma pass rates were 98.7% ± 1.2% for prostate cancer patients and 96.2% ± 2.1% for lung cancer patients, using a 10% dose threshold. Although all prostate cases were reconstructed using iCBCT MAR to avoid metal artifacts of gold fiducials, the impact of various metal implants in real patient anatomies on dose calculation accuracy was not tested directly. Consistent with these prior findings, our results demonstrated similarly high gamma pass rates and low DVH deviations for HyperSight images, supporting its robust dosimetric performance across anatomical sites and suitability for direct use in adaptive radiotherapy.

A study by Nelson et al. dosimetrically analyzed a CTsim‐optimized IMRT plan calculated on a HyperSight image of an H&N anthropomorphic phantom,^40^ The dose differences from CTsim across all contours evaluated were within 0.5% for the mean dose and within 3% for the D98% and D2% metrics. This work only evaluated the standard HyperSight iCBCT Acuros reconstruction for the H&N anthropomorphic phantom. Additionally, their H&N phantom did not contain high‐density dental implants producing metal artifacts, such as those observed in our patient data. Real patient images introduce additional complexities that cannot be fully replicated by phantoms, including heterogeneous tissue composition, clinically relevant metallic implants, and variability in patient anatomy.

One limitation of this study is that our CTsim FBCT did not include a MAR reconstruction option. While MAR algorithms are available on some CT simulators, the FBCT images were reconstructed without MAR, reflecting standard clinical practice at our institution, even though 80% of patients exhibited metal‐induced artifacts. To address this, artifacts were contoured on the FBCT images and manually overridden to an HU of 0 g/cm^3^. This approach was used to produce a reference within the limitations of our available FBCT system, allowing a comparative evaluation of dosimetric differences attributed to the CBCT images. Previous studies have demonstrated that advanced MAR techniques in FBCT can improve image quality and HU accuracy, necessary for reliable delineation of targets and OARs during treatment planning.^41^, ^42^, ^43^ In addition, comparisons between manual density override methods and MAR‐based reconstructions have shown that MAR approaches result in dose calculations that are closer to reference conditions without metal implants than those obtained using HU override techniques.^44^, ^45^ Therefore, while the manual override approach used here is consistent with institutional workflow, it does not fully represent an optimal approximation of ground truth in the presence of metal artifacts. Any observed deviations from reference conditions may reflect limitations of this FBCT correction method in addition to differences between FBCT and CBCT imaging. Future studies could examine how dosimetric distributions differ between CTsim FBCT images with MAR reconstruction and HyperSight iCBCT MAR images.

Another important consideration concerns the timing of image acquisition. HyperSight CBCTs were acquired during the same session as the FBCT, minimizing anatomical differences between scans, while TrueBeam CBCTs were obtained at the first treatment fraction, 12 to 20 days after FBCT acquisition. In H&N radiotherapy, time‐dependent anatomical variations are associated with treatment‐induced weight loss, soft tissue shrinkage, and tumor response, which typically develop progressively during the treatment course and after, rather than over short time intervals prior to treatment initiation.^46^, ^47^, ^48^ This assumption was supported by qualitative review, with minimal anatomical variation observed at the pre‐treatment stage across all patients. The analyzed regions of interest were located away from areas affected by day‐to‐day positioning variations, such as skin folds behind the neck, which were likely caused by mask setup or shoulder positioning. Future research will focus on validating the clinical use of HyperSight images through a qualitative image assessment and the delineation and verification of critical organs. These efforts are critical for clinically implementing HyperSight into adaptive radiotherapy workflows.

## 5 CONCLUSIONS

This study demonstrates the feasibility of HyperSight CBCT for direct dose calculations in H&N adaptive radiotherapy. The MAR reconstruction provided the highest dose accuracy in regions affected by metal artifacts, while the Acuros reconstruction exceled in the absence of artifacts. These findings highlight the importance of selecting reconstruction modes based on patient‐specific conditions to ensure dosimetric reliability. TrueBeam CBCT exhibited lower accuracy in artifact‐prone areas, however all evaluated CBCT systems produced DVH metric deviations within clinically acceptable limits for the PTV and OARs. Overall, our findings demonstrate that direct dose calculation on HyperSight CBCT is accurate and supports adaptive planning, even for patients with high density implants.

## CONFLICT OF INTEREST STATEMENT

Amanda Cherpak and Lee MacDonald are members of the Intelligent Imaging Consortium (IIC) with Varian. After completion of the work reported in this manuscript, Amanda Cherpak became an employee of Varian. All other authors have no conflicts of interest to declare.

## Data Availability

Access to unpublished data from our study is subject to negotiation with the study sponsor, Varian Medical Systems, Inc., or as may be required by applicable law.
